# Pathological complete remission of a locally advanced gastric cancer by neoadjuvant therapy “sandwich” regimen as SOXAP+ fluorescence laparoscopic surgery +SOXAP: Case report

**DOI:** 10.3389/fphar.2022.1008755

**Published:** 2022-11-02

**Authors:** Yanling Ma, Bofang Wang, Ewetse Paul Maswikiti, Xueyan Wang, Na Wang, Hao Chen

**Affiliations:** ^1^ Second Clinical Medical College, Lanzhou University, Lanzhou, Gansu, China; ^2^ Department of Cancer Center, Lanzhou University Second Hospital, Lanzhou, China

**Keywords:** locally advanced gastric cancer, oxaliplatin, S-1, apatinib, camrelizumab, complete remission

## Abstract

Gastric cancer is an extremely burdensome and challenging malignant tumor with a high incidence and a high mortality rate, which seriously results in a thorny prognosis for oncology patients. Surgical treatment combined with postoperative adjuvant therapy are currently the most regular methods for the treatment of locally advanced gastric cancer (LAGC), but long-term efficacy is not an ideal outcome. Therefore, herein we report a case of a pathologically confirmed complete remission of LAGC treated by the administration of neoadjuvant therapy combined with fluorescence laparoscopic surgery with more significant long-term survival. With that being mentioned, a 60-year-old man was diagnosed as moderately differentiated gastric antrum adenocarcinoma (T3N1M0). Moreover, after three cycles of SOXAP regimen (Oxaliplatin + S-1+Apatinib + Camrelizumab), and it was found out that the gastric lesion was smaller in size than before, total laparoscopic radical resection of the distal gastric cancer was performed at the time. Furthermore, no tumor cells were seen in gross specimen post operatively, achieving complete remission of the case. In addition, he also underwent three cycles of SOXAP regimen postoperatively. Interestingly and assuredly, he was in good health after an almost 2-year follow up period. These results suggest that this therapeutic regimen is a promising treatment modality for the management of locally advanced gastric cancers.

## Introduction

Gastric cancer (GC) is the fifth most common cancer in the world and the fourth cause of cancer-related deaths (Sung et al., 2021). Due to its atypical early symptoms and insufficient screening strategic approach, about 70% of gastric cancer patients in China are already in their locally advanced stage when the final diagnosis is formulated (Smith et al., 2005; Petrillo et al., 2019). In addition, radical surgery is the most essential treatment strategy for locally advanced gastric cancer, but surgical treatment alone and traditional surgery combined with postoperative adjuvant treatment have been found to be ineffective. Postoperative patients are prone to have some recurrence and metastasis, with a low 5-year survival outcomes. However, in these recent years, the “sandwich method”, namely, neoadjuvant chemotherapy + surgery + postoperative adjuvant therapy has achieved relatively better outcomes and benefits compared with the previous ones. Nevertheless, the disease control rate and conversion success rate of simple neoadjuvant chemotherapy is still very limited, for example DCF regimen 34% (Ozdemir et al., 2014), and the pathologic complete response (pCR) is relatively low; DCF regimen 9%,ECF 6%,FLOT regimen less than 20% (Ferri et al., 2012; Al-Batran et al., 2016). Apatinib is currently the third-line treatment drug for advanced gastric cancer recommended by the CSCO guidelines, and the study of Zheng et al. showed that the total pathological effective rate of Apatinib combined with SOX chemotherapy for neoadjuvant treatment of gastric cancer was as high as 89.7% (Zheng et al., 2020). And there is a clinical trial proved that oxaliplatin and capecitabine combined with Apatinib used as new adjuvant therapy, showing good efficacy and manageable safety in patients with LAGC or GEJ (Tang et al., 2022). Zhi et al. reported a clinical trials result, Camrelizumab Combined with Chemotherapy Followed by Camrelizumab plus Apatinib as First-line Therapy for Advanced Gastric or Gastroesophageal Junction Adenocarcinoma, which can make GC patients gain FPS and OS benefits, moreover this regime has a manageable toxicity (Peng et al., 2021). therefore, in this case, targeted therapy and immunotherapy were utilized and neoadjuvant therapy as well. Moreover, the RESOLVE study indicated that the 3-year follow-up of progression-free survival of SOX regimen + D2 resection + SOX regimen is 62.02% (*p* = 0.045), the results were higher than those of the other two groups, suggested SOX as a new adjuvant therapy for gastric cancer is a good scheme (Zhang et al., 2021). Herein, we report a case of a locally advanced gastric cancer patient who had a pathologically CR by neoadjuvant therapy(Oxaliplatin + S-1 + Apatinib + Camrelizumab)confirmed in a gross specimen after total laparoscopic radical resection of distal gastric region under fluorescence laparoscopy.

## Patient’s clinical information

**FIGURE 1:**
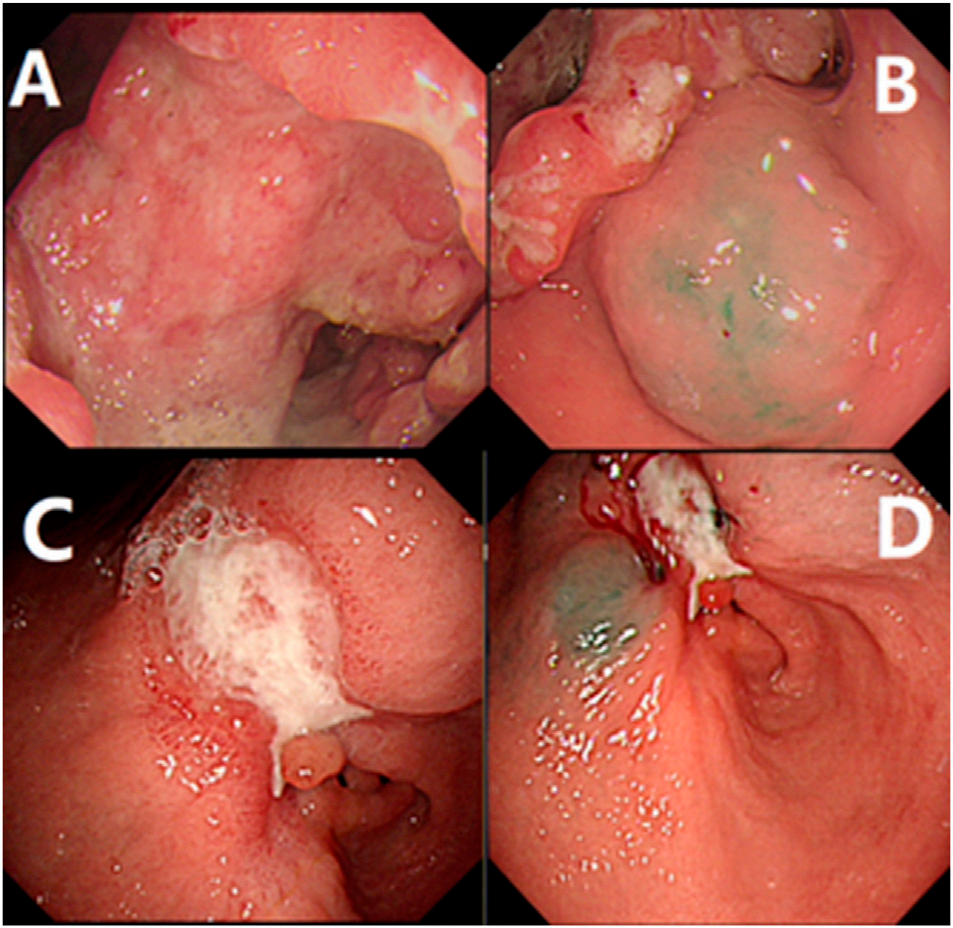
Gastroscopy examination and indocyanine green injection. **(A)** showed the result of electronic gastroscopy before treatment, showing an ulcerous lesion in the gastric antrum, about 6*7 cm in size, gastric cancer (Borrmann III type), **(B)** showed indocyanine green was injected submucosally at the edge of the lesion before treatment; **(C)** showed the result of electronic gastroscopy after neoadjuvant treatment, the size is about 3*2 cm, gastric cancer (Borrmann type II), the lesion is smaller than the previous. **(D)** showed indocyanine green is injected as before after neoadjuvant treatment.

**FIGURE 2:**
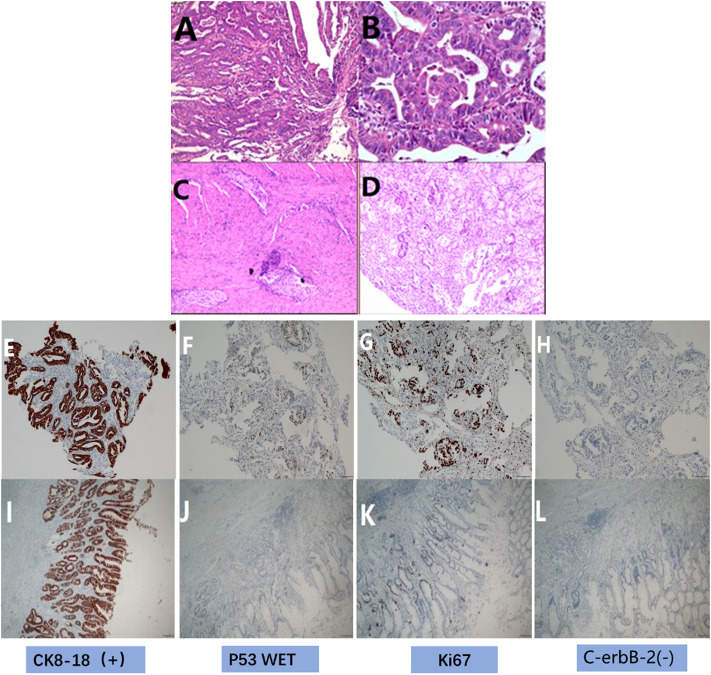
HE and immunohistochemical staining. **(A)** HE before treatment showed medium-poorly differentiated adenocarcinoma (original magnification ×100), **(B)** HE before treatment showed medium-poorly differentiated adenocarcinoma (original magnification ×400), **(C)** HE after postoperative showed there were many proliferations of lymphocytes and foam cells, no clear tumor cells were seen(original magnification ×100). **(D)** HE after treatment showed there were many proliferations of lymphocytes and foam cells, no clear tumor cells were seen(original magnification ×400). **(E)** IHC before treatment showed the expression of CK8/18 (+), **(F)** IHC before treatment showed the expression of p53 (wild type expression), **(G)** IHC before treatment showed the expression of Ki67 positive cell number (90%+), **(H)** IHC before treatment showed the expression of C-erbB-2 (0); **(I)** IHC after postoperative showed the expression of CK8/18 (+), **(J)** IHC after postoperative showed the expression of p53 wild type, **(K)** IHC after postoperative showed the expression of the number of Ki67 positive cells (10%+), **(L)** IHC after postoperative showed the expression of C-erbB-2 (0).

**FIGURE 3:**
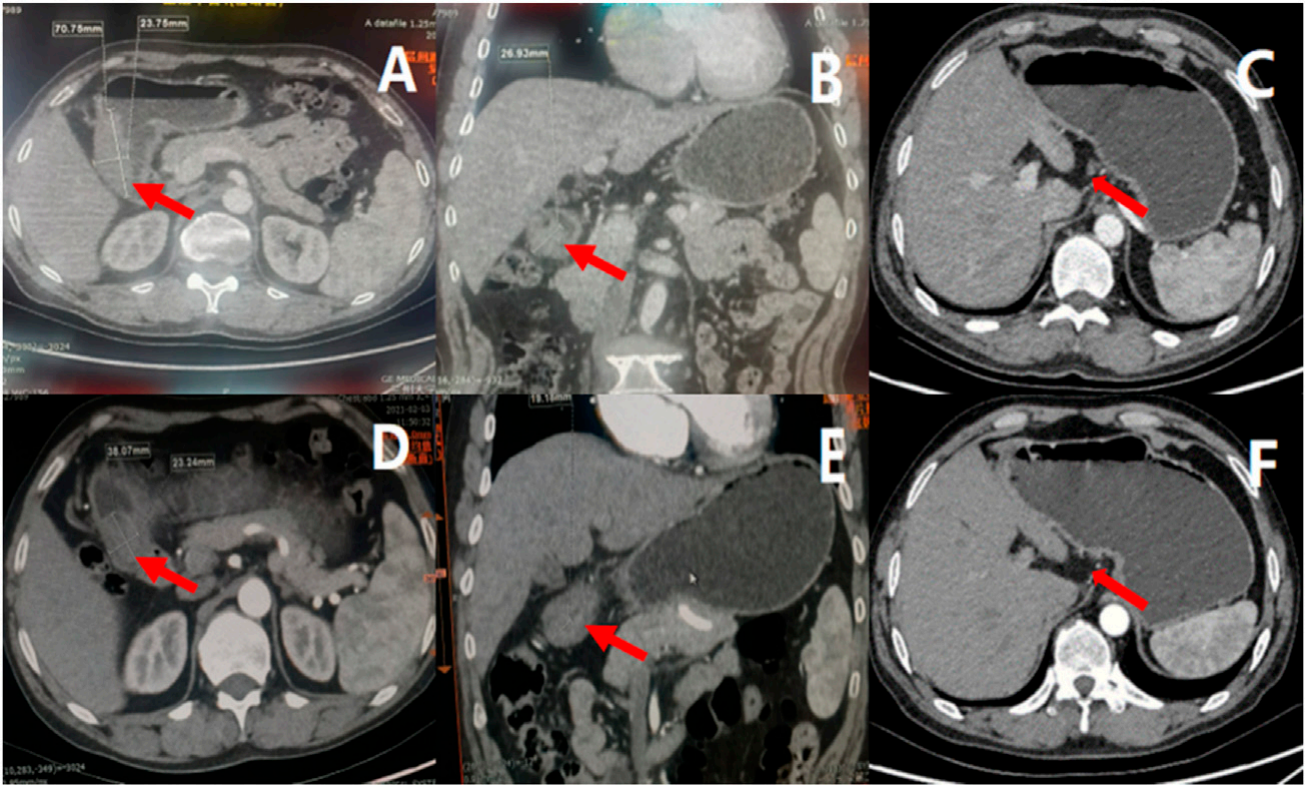
Results of enhanced CT before and after neoadjuvant therapy, indicated tumor and lymph node volume decreased after three cycles of treatment. **(A)** showed the cross section of gastric antrum tumor before treatment, **(B)** showed the coronal view of gastric antrum tumor before treatment, **(C)** showed the enlarged lymph nodes before treatment; **(D)** showed the cross section of gastric antrum tumor after neoadjuvant treatment, **(E)** showed the coronal view of gastric antrum tumor after neoadjuvant treatment, **(F)** showed the enlarged lymph nodes after neoadjuvant treatment.

A 60-year-old male patient was admitted to the hospital with a history of “abdominal pain and discomfort for 1 month, aggravation and fatigue for a week.” He has had a history of hypertension for 7 years to date, during which he took antihypertensives (indapamide tablets, 2.5 mg, qd, PO), and his blood pressure was now well controlled. In addition, the general health condition was fair, mild anemia, mild upper abdominal tenderness, no rebound pain and muscle tension. Collectively, gastroscopy and biopsy at his local hospital where he first sought consultation showed gastric cancer and moderately differentiated adenocarcinoma. On the contrary, gastroscopy in the author’s hospital revealed a gastric cancer at the antrum with an ulcerative lesion of an approximate surface area of 6 cm*7 cm (Bormann III type) **(** Figure 1A **)**, and the biopsy result at the time of consultation by the department of pathology showed a moderate to a poorly differentiated adenocarcinoma. Moreover, immunohistochemical staining demonstrated cancer cells: CK8/18 (+), p53 (wild type expression), C-erbB-2 (0), Syn (-), CgA-, CD56^−^, p504s-, Ki67 positive cells (90%+) and EREB (-), (Figures 2A,B,E–H). On the contrary, tumor markers results were as follows: AFP 2.18 ng/ml, CEA 4.01 ng/ml, CA125 5.05 u/ml, CA199 15.5 u/ml, CA72-4 1.5 u/ml. HER2 (-) EB-DNA (-), HP (-). An abdominal enhanced CT indicated a thickening of the stomach wall in the antrum (Maximum thickness 26.93 mm), multiple enlarged lymph nodes in the hepato-gastric space **(** Figures 3A–C). This patient’s ultimate final clinical diagnosis was formulated to be gastric cancer with clinical stage T3N1M0 (stage IIb), pathological diagnosis of moderate to poorly differentiated adenocarcinoma. PS:1 point; NRS: two points.

**FIGURE 4:**
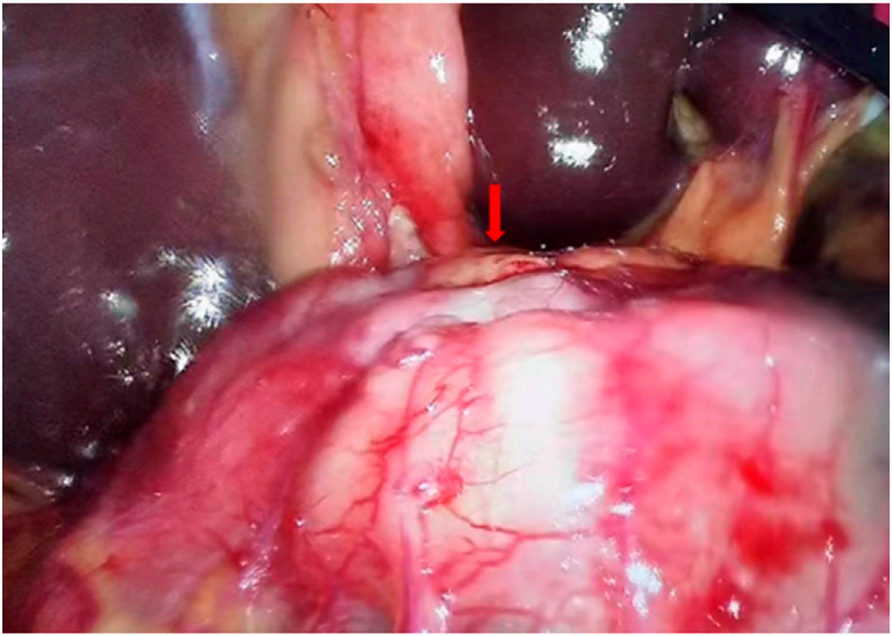
Laparoscopy exploration indicates visible tumor lesions, obviously at least penetrated the serosal layer.

Collectively, following Multidisciplinary team (MDT) intense and in-depth discussions, it was decided that abdominal exploration with the fluorescence laparoscopic surgery be performed to the patient. In addition, indocyanine green injection along the edge of the lesion in four directions/dimensions (2.5 mg/ml, 250 ul) respectively was administered under the guidance and visualization aid of a gastroscope prior to surgery **(** Figure 1B **)**. Notably, an intraoperative exploratory procedure revealed that the patient’s lesion had obviously penetrated the serosal layer **(** Figure 4 **)**, considering a clinical stage at least T4aN1M0(stage IIIa). Contrarily, neoadjuvant treatment was recommended after explaining and health education of the patient’s household family members. Following yet another MDT discussion session, it was decided to perform the SOXAP program (oxaliplatin + S-1 + Apatinib + carrelizumab) as neoadjuvant treatment and then surgery, oxaliplatin (Laboratories Thissen S.A Sanofi Winthrop Industries) 130 mg/m2, d1, ivgtt; S-1 (Taiho Pharmaceutical Co., Ltd. Company), 60 mg/m2, bid, d1-d14, pro; Apatinib mesylate (Jiangsu Hengrui Pharmaceutical Co., Ltd.) 250 mg, qd, d1-d30, pro; Carrelizumab (Jiangsu Hengrui Pharmaceutical Co., Ltd.) 200 mg, d1, ivgtt, in every 3 weeks which was a complete cycles. During undergoing treatment procedure, he experienced discomforts and adverse digestive tract reactions, such as intermittent nausea, retching, poor appetite and leukopenia(WBC 2.87 x 10∼^9^/L, which returned to normal ranges after being treated by a recombinant human granulocyte macrophage stimulating factor 2 ug/kg) **(** Table 1 **)**,. In addition, tumor markers, thyroid function, liver function, pancreas function **(** Tables 2, 3, 4 **)**, were all basically within the normal range, and there was no significant change observed as in abnormalities detected during the treatment course.

**TABLE 1:** Changes of main indexes of blood routine during neoadjuvant therapy cycle.

	WBC(10–9/L)	RBC(10–12/L)	HGB(g/L)	PLT(10–9/L)
FC	3.65	4.82	91	327
SC	5.26	4.73	99	227
TC	2.87	4.55	102	109

FC, first circle; SC, second circle; TC, third circle.

**TABLE 2:** Thyroid function changes during neoadjuvant therapy cycles.

	T3(nmol/L)	T4(nmol/L)	FT3(pmol/L)	FT4(pmol/L)	TSH(uIU/ml)	TG (ng/ml)	TgAb(U/ml)	TPOAb(U/ml)
FC	1.35	114.1	4.88	17.28	3.15	15.5	20.7	<28
SC	1.94	104.1	4.88	11.65	8.13	26	16.9	35.4
TC	1.76	116.9	4.29	12.83	12.638	36.2	<15	41.1

FC, first circle; SC, second circle; TC, third circle.

**TABLE 3:** Changes in liver function during neoadjuvant therapy cycles.

	TBIL (μmol/L)	DBIL (μmol/L)	IBIL (μmol/L)	ALT (U/L)	AST (U/L)	γ-GT (U/L)	ALP (U/L)	TB (g/L)	ALB (g/L)	GLB (g/L)	AMY (U/L)
FC	14.6	7.6	7.0	50	16	22	59	67.9	40.5	27.4	69
SC	11.9	4.6	7.3	14	18	18	79	68.7	38.7	30	110
TC	19.4	8.4	11	21	31	22	101	70.1	39.2	30.9	106

FC, first circle; SC, second circle; TC, third circle.

**TABLE 4:** Changes in tumor markers during neoadjuvant therapy cycles.

	AFP(ng/mL)	CEA(ng/mL)	CA125(U/mL)	CA199(U/mL)	CA72-4(U/mL)
FC	1.73	2.02	12.60	6.41	24.30
SC	3.26	2.34	7.65	10.50	4.18
TC	3.59	2.59	6.52	5.88	<1.5

FC, first circle; SC, second circle; TC, third circle.

After three cycles of treatment, the drug was terminated and withheld for 1 month, and the gastroscopic procedure was reperformed. Strikingly, there was an ulcerous lesion in the gastric antrum measuring a surface area of about 3*2 cm, which significantly reduced to at least a half as compared to before he underwent treatment, and the depth of the ulcers were significantly reduced, **(** Figure 1C **)**. On the other hand, a contrast-enhanced CT-scan of the abdomen after three circles of treatment revealed a thickness (maximum thickness 19.18 mm) and a volume of the gastric antrum to have been reduced compared to the previous one, and the number and size of enlarged lymph nodes around the cardia, left side of stomach and hepato-gastric space were significantly reduced as compared with those prior to undertaking treatment, (Figures 3D–F). According to the RECIS scoring standard, the overall therapeutic effect evaluation on the lesion reached PR. Therefore, in MDT sessions and series of discussions it was suggested that radical resection of distal gastric cancer be performed along with fluorescence laparoscopy.

**FIGURE 5:**
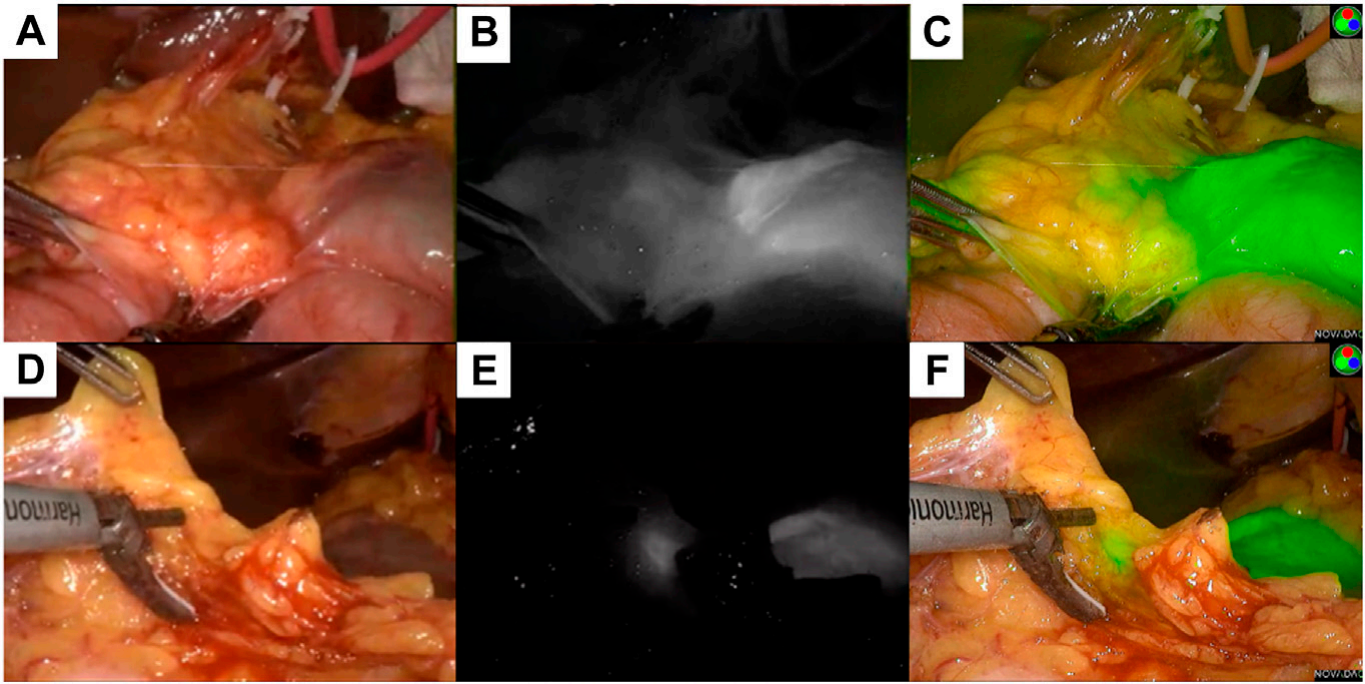
Different mode of the lesion under fluorescence laparoscopy during the radical operation. **(A)** showed the normal mode of a primary lesion, **(B)** showed the black and white mode of the primary lesion, **(C)** showed the fluorescence mode of the primary lesion; **(D)** showed the normal mode of a lymph node lesion, **(E)** showed the black and white mode of the lymph node lesion, **(F)** showed the fluorescence mode of the lymph node lesion.

**FIGURE 6:**
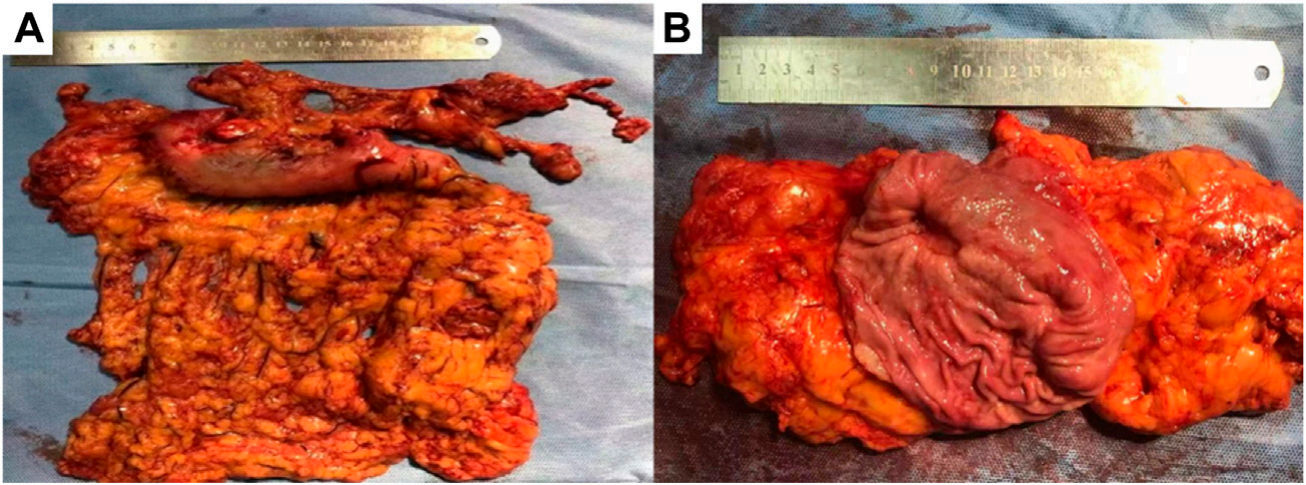
Gross specimen after resection, **(A)** showed the exterior view without serosa invasion, **(B)** showed the interior view, gastric mucosal ulcer, about 2*1.5 cm in size, without breaking through the serosa.

**FIGURE 7:**
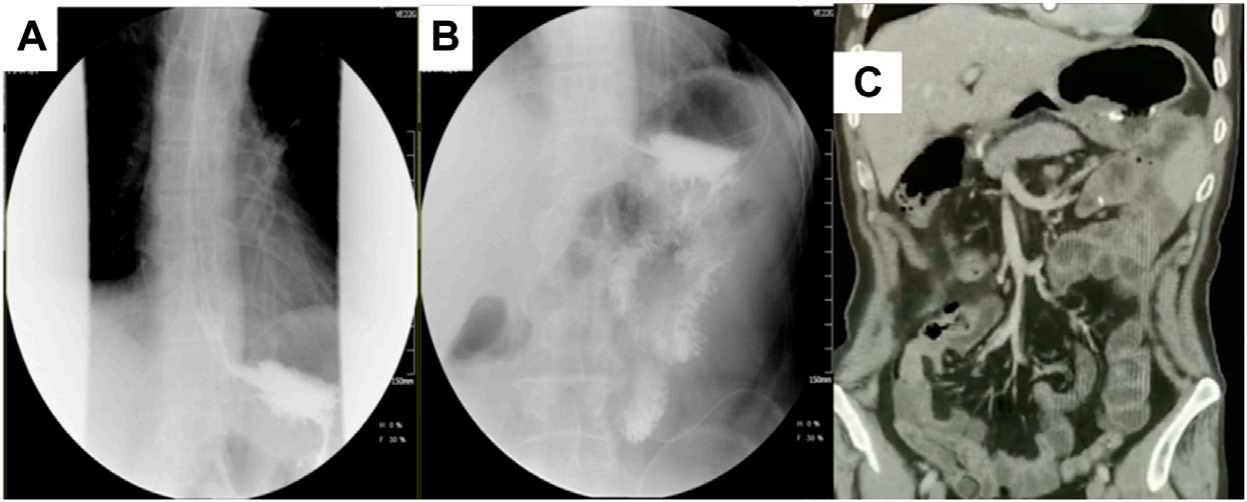
Postoperative upper gastrointestinal angiography and CT coronal plane. **(A,B)** showed that the reconstructed digestive tract is unobstructed, and the anastomosis is normal. **(C)** showed no obvious signs of recurrence and metastasis after surgery and another three cycles of adjuvant treatment.

Indocyanine green was injected and administered into the submucosa as before **(** Figure 1D **)**. During fluorescence laparoscopic exploration, there was no obvious exudation and abnormalities in the abdominal and pelvic cavity. The lesion was located at the corner of the stomach in the fluorescent laparoscopic green development mode without serosa invasion **(** Figures 5A–F). Then, it was decided that total laparoscopic radical distal gastrectomy + Roux-en-Y + uncut + Braun anastomosis be performed. The procedure was uneventful, lasting 150 min with 50 ml recorded as intraoperative blood solution. Furthermore, postoperative specimens were pictured/photographed and revealed gastric corner ulcerous lesions, without abnormalities in the external appearance of the serosal layer, **(** Figures 6A,B). Post-surgery, the patient recovered remarkably, and could ambulate and walk around the next day without any marked discomforts. Moreover, his intestinal peristaltic movements and bowels got back to normal and were recovered on the third day post-surgery. In addition, angiographic examination of the upper gastrointestinal tract showed that the reconstructed gastrointestinal tract was unobstructed, and the anastomotic stoma was normal, as evidenced by the contrast agent going through smoothly and unobstructed (Figures 7A,B). He was discharged on the seventh day. Postoperative pathological examination illustrated distal gastrectomy specimen: stomach angle mucosal ulcer, among which there were many lymphocytes and foam cell proliferation, combined with immunohistochemical staining, no clear tumor cells were observed, tumor regression grade (Becker grade: Grade 1a). Additionally, there was no invasion in the nerves and vessels, and no residual cancer tissues were observed on both sides of the incisional margin. Collectively, the mucosa around the ulcer showed mild changes in atrophic gastritis. The lymph node test results were as follows: Lymph nodes of lesser curvature of the stomach (0/10), Lymph node of greater curvature of the stomach (0/9), no cancer metastasis was found. Furthermore, lymph nodes were sent separately) (0/39), no cancer metastasis; group 1 (0/1), group 3 (0/1), group 4 (0/9), group 6 (0/7), group 7 (0/7), nine groups (0/7), 10 groups (0/4), 11 groups (0/2), 12 groups (0/1). Immunohistochemical staining: glands revealed: CK8/18(+), CKp (+), p53(-), C-erbB-2(0), PMS-2 (+), MLH-1 (+), MSH- 6 (+), MSH-2 (+), Syn (-), Ki67 positive cells number 10%, **(** Figures 2C,D,I–L). It could be seen that these gene expressions were consistent with those before treatment, and Ki67 was significantly reduced.

**FIGURE 8:**
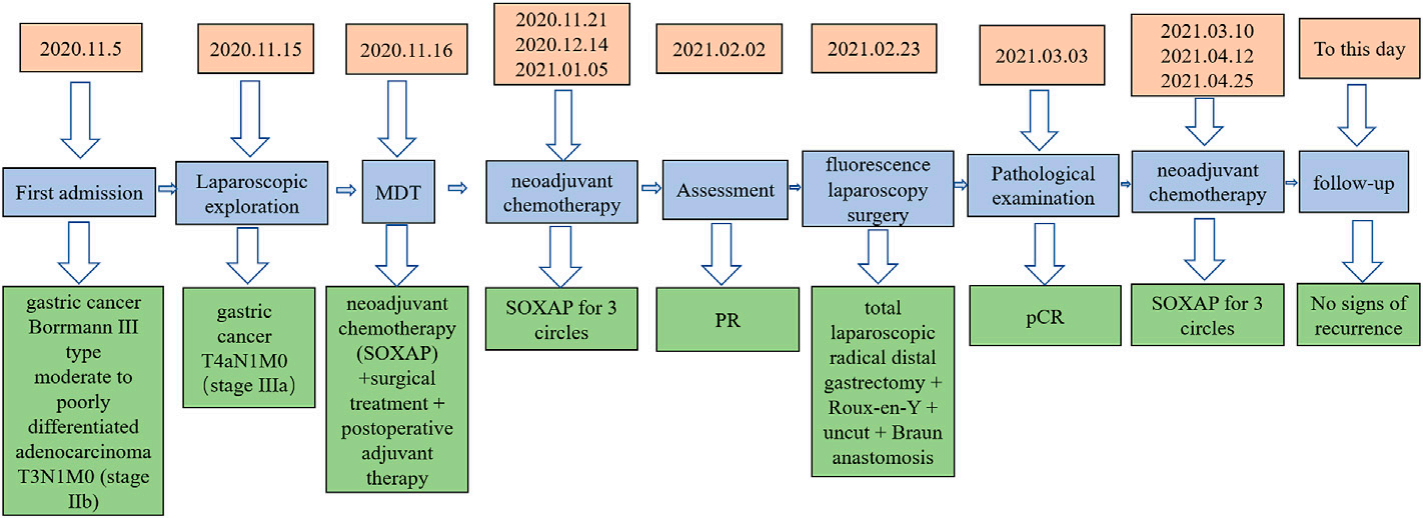
The timeline of the patients’ treatment.

One month following operation, the circulating tumor cell test (CTC) revealed that there were two active tumor cells in the blood. On the contrary, the original dose of SOXAP therapy was continued for three cycles. After that, the CT coronal plane showed no signs of disease recurrence (Figure 7C), and chemotherapy was terminated. Followed up for almost 2 years, during which the patient occasionally felt some nausea, and reflux, released post symptomatic treatment. The timeline of the patients’ treatment is described in Figure 8.

## Discussion

According to the Global cancer statistics 2020: GLOBOCAN estimates of incidence and mortality worldwide for 36 cancers in 185 countries, GC in world ranks the fifth in the global cancer incidence and fourth in the occurrence of mortality rates (Sung et al., 2021)^.^ Nonetheless, surgery is the only possible and first choice curative therapeutic strategy, especially in early local disease manifestation. In addition, with limitation of the lesion to the mucosa and submucosa, a 5-year survival rate of about 90%, can be achieved by surgical approach alone (Nashimoto et al., 2003). Unfortunately, most patients are often diagnosed at an advanced stage with an already worsened thorny prognosis. When the tumor penetrates through the submucosa, a 5-year survival rate may be as low as 20%–30% (Roukos et al., 1995). Previously surgery was still the best therapeutic option for advanced gastric malignancies with a poor prognosis. On the contrary, this patient has undergone an imaging evaluation examination and considered a clinical stage T3N1M0. Initially, surgical treatment was recommended. However, after laparoscopic exploration, it was found that the patient’s tumor had at least penetrated the serosal layer, clinical stage T4aN1M0. In addition, he had to undergo neoadjuvant therapy according to the NCCN Clinical Practice Guidelines in Oncology of Gastric Cancer (Ajani et al., 2022). Post detailed health education and discussions, his household family members agreed to that he should undergo neoadjuvant treatment prior to radical surgery.

Notably, neoadjuvant therapy is a comprehensive treatment performed prior to surgery. Collectively, its advantages are reduction in tumor staging phenomenon, lowering of primary tumor burden, eliminating tumor micro-metastasis, downcutting intraoperative spread and postoperative recurrence, and increasing the resection rate of gastric cancer (Das, 2017). On the other hand, chemotherapy is the current main method and part of neoadjuvant therapy. In 1982, Frei first proposed the concept of neoadjuvant chemotherapy (Frei 3rd, 1982). In 2006, the MAGIC study randomly divided 503 patients with gastric or gastroesophageal junction and low-segment esophageal adenocarcinoma into two groups. The final results showed that compared with surgery alone, surgery combined with ECF (Epirubicin + cisplatin + fluorouracil) chemotherapy group could significantly improve the 5-year survival rate (36.3% and 26%, *p* < 0.01) (Cunningham et al., 2006). Subsequently, in 2007, the FNCLCC-FFCD study showed that compared with surgery alone, CF regimen (cisplatin + fluorouracil) neoadjuvant chemotherapy combined with surgery can significantly increase the R0 resection rate of patients with gastric cancer (84% and 73%, *p* = 0.04), 5-year overall survival rate (38% and 24%, *p* = 0.02) and 5-year disease-free survival rate (34% and 19%, *p* = 0.02) (Ychou et al., 2011). Although these two studies lack accurate clinical staging for the enrollees, these two studies have fully established the status of neoadjuvant chemotherapy in the treatment of gastric cancer.

Japan’s JCOGO405 single-arm experiment proved that, S-1 combined with cisplatin before surgery for two to three cycles before radical gastric cancer (D3 lymph node dissection), R0 resection rate was 82.35%, 5-year overall survival and 5-year recurrence-free survival was 53% and 50%(Yoshikawa et al., 2009), respectively. In 2019, ESEO announced the results of a 3-year follow-up of the RESOLVE study in China. The study divided 1094 patients (cT4aN + M0 or cT4bNxM0) into three groups randomly and received D2 surgery + XELOX regimen (capecitabine + oxaliplatin), D2 surgery + SOX regimen (S-1+ oxaliplatin) and SOX regimen + D2 resection + SOX regimen, respectively, 3 years of progression-free survival rates was 54.78%, 60.29%, and 62.02% (*p* = 0.045) (Zhang et al., 2021). Based on the results of this study, SOX may become the first choice for perioperative chemotherapy for gastric cancer in China.

Compared with surgery alone, the combination of neoadjuvant chemotherapy did increase the R0 resection rate,OS and DFS of patients, However, chemotherapy was less effective for gastric cancer than for other solid malignancies due to tumor heterogeneity.The results of a Phase II NEOTAX study showed that the objective response rate of DCF regimen as neoadjuvant chemotherapy for gastric cancer was only 34% (Ozdemir et al., 2014), with a pathological complete remission rate after neoadjuvant chemotherapy DCF regimen and ECF regimen of only 9% and 6%. Besides, the results illustrated that some patients had a higher incidence of adverse drug reactions, and only 51% of the patients completed the treatment regimen (Ferri et al., 2012; Al-Batran et al., 2016). Since the side effects of patients after chemotherapy alone were obvious and evident, it leads to a delay in the treatment cycle of patients, reducing the overall treatment effect. Therefore, there is an urgent need for new, effective treatment regimens with acceptable safety profiles.

Targeted therapy, as one of the effective methods for the treatment of locally advanced gastric cancer, has also been continuously applied to neoadjuvant therapy. However, trastuzumab, an anti-Her-2 positive antibody that was first used in gastric cancer, has been proven to have some advantages over chemotherapy alone (Bang et al., 2010). Clinical trials of trastuzumab combined with neoadjuvant chemotherapy are still underway, including the INNOVTION study in South Korea and NCT02205047 in the Netherlands, and the JCOG1301 study registered as UMIN000016920 in Japan. Furthermore, the results are worth looking forward to. Anti-vascular drugs are also effective targeted drugs widely used in advanced gastric cancer. Ma et al. reported that compared with DOF regimen (docetaxel + oxaliplatin + fluorouracil), bevacizumab combined with DOF regimen as neoadjuvant chemotherapy had a significantly higher R0 resection rate in patients with gastric cancer (75% and 50%, *p* = 0.0209), disease-free survival time was significantly prolonged (15.2 and 12.3 months, *p* = 0.013), and increased delayed wound healing and incidence of complications such as anastomotic leakage (Ma et al., 2015). Contrarily, Apatinib is currently the third-line treatment drug for advanced gastric cancer recommended by the CSCO guidelines. A clinical study showed that the total pathological effective rate of alpatinib combined with SOX chemotherapy in the neoadjuvant treatment of gastric cancer was 89.7% (Zheng et al., 2020). Therefore, in this case, Apatinib was chosen as one of the therapeutic drugs of choice for the neoadjuvant therapy.

Immunotherapy plays on the other hand with some anti-tumor effects by activating the immune system and relieving immune suppression. The ATTRACTION-2 study showed that compared with placebo, PD-1 monoclonal antibody can significantly improve the median survival time of patients with advanced gastric cancer or esophagogastric junction adenocarcinoma (5.26 and 4.14 months, *p* < 0.01), with that 1 year overall survival rates were 27.3% and 11.6%, and the 2-year overall survival rates were 10.6% and 3.2%, respectively, indicating that patients with advanced gastric cancers could benefit from PD-1 monoclonal antibody therapies (Chen et al., 2020). Although there is no uniformity in the clinical stages of patients in these clinical trials, the overall treatment still has good benefits (Jin et al., 2020; Zhang et al., 2020). Moreover, studies have shown that after neoadjuvant chemotherapy, the overall expression of PD-1 and PD-L1 in gastric cancer is up-regulated (Yu et al., 2019), which means that neoadjuvant chemotherapy, may improve the response rate of gastric cancer patients as compared to the administration of immunotherapies.

Based on the above research results, the “sandwich method” namely, neoadjuvant chemotherapy + surgical treatment + postoperative adjuvant therapy was utilized in this case, preoperative SOXAP for three circles + D2 surgery + postoperative SOXAP for three circles. Interestingly, the patient had no tumor cells in the removal of the distal stomach and removal of lymph nodes, achieving a remarkable pCR.

Some studies have shown that patients with advanced gastric cancer who reach pCR after receiving neoadjuvant chemotherapy have a very high overall survival rate and recurrence-free survival rate, which could improve the prognostic status of patients at an early stage of gastric cancer (Cho et al., 2015). After regular treatment in this case, the pathological complete remission was finally achieved and remarkable. However, due to the patient’s economic status, genetic testing and sequencing technological tests were not performed in the detection of the patient’s genetic changes. Therefore, the genes or factors related to the pathological complete remission were not clearly and vividly stated.

However, we should not only focus on the efficacy of the treatment regimen, but also on its safety. According to some previous reports, hypertension, hand-foot syndrome, and proteinuria are the most common adverse effects of antiangiogenic drugs. In order to clarify the efficacy and safety of Apatinib combined with SOX in the treatment of locally advanced gastric cancer, Liu et al. conducted a multicenter, prospective, phase 2 trial and found the most common adverse reactions of Apatinib combined with SOX to be neutropenia, leukopenia, elevated transaminases, and anemia. Collectively, these data demonstrated that the toxic effects of Apatinib combined with SOX are mainly SOX-related toxic effects. Most toxicities were grade 1 or 2, without chemotherapy-related death, adverse effects and serious threatening complications. Therefore, Apatinib combined with chemotherapy is well tolerated (Lin et al., 2021). Notably, there is no definite data on the efficacy and safety of chemotherapy combined with targeted therapy and immunotherapy in the treatment of locally advanced gastric cancer. It is hoped that further clinical trials would give rise to more reliable data with auspicious outcomes.

In summary, neoadjuvant chemotherapy can increase the effect of immunotherapy to a greater extent. In addition, targeted therapy and immunotherapy have some synergism. In conclusion, chemotherapy combined with targeted therapy and immunotherapy as new adjuvant therapeutic strategies will assuredly benefit patients with gastric cancer at advanced stages. However, there is still need for elucidation and therapeutic efficacies through clinical trials to prove this phenomenon.

## Data Availability

The original contributions presented in the study are included in the article/supplementary material, further inquiries can be directed to the corresponding author.

## Abbreviations

CF, cisplatin + fluorouracil; CSCO, chinese society of clinical oncology; CT, computed tomography; CTC, circulating tumor cell; DOF, docetaxel + oxaliplatin + fluorouracil; ECF, Epirubicin + cisplatin + fluorouracil; GC, gastric cancer; LAGC, locally advanced gastric cancer; MDT, multi-disciplinary treatment; NRS, nutritional risk screening score; pCR, complete pathological remission; PD-1, programmed cell death-1; PD-L1, programmed cell death-Ligand 1; PS, performance status; SOXAP, oxaliplatin + S-1+apatinib + camrelizumab; XELOX, capecitabine + oxaliplatin.
